# Experience of financial toxicity and coping strategies in young and middle-aged patients with stroke: a qualitative study

**DOI:** 10.1186/s12913-023-10457-z

**Published:** 2024-01-17

**Authors:** Ling Xu, Qiong Dong, Aiping Jin, Sining Zeng, Kai Wang, Xiaopei Yang, Xiaoping Zhu

**Affiliations:** 1 Department of Nursing, Shanghai Tenth People’s Hospital, Tongji University School of Medicine, Shanghai, China; 2 Department of Neurology, Shanghai Tenth People’s Hospital, Tongji University School of Medicine, Shanghai, China; 3 School of Nursing, Anhui University of Chinese Medicine, Hefei, Anhui China

**Keywords:** Stroke, Financial toxicity, Psychological pressure, Coping strategies, Qualitative study

## Abstract

**Background:**

While financial toxicity (FT) is prevalent in patients with cancer, young and middle-aged patients with stroke are also affected by FT, which can exacerbate their physical and psychological challenges. Understanding the patient’s experience and response measures can further understand the impact of FT on patients with stroke, to help alleviate FT. However, little is known concerning the experience of patients with stroke with FT or their coping strategies. Therefore, this study aimed to describe the experiences of FT in young and middle-aged patients with stroke and their coping strategies.

**Methods:**

A phenomenological method was utilized. Semi-structured interviews were conducted with 21 young and middle-aged stroke patients (aged 18–59) between October 2022 and March 2023. The participants were recruited from a tertiary hospital in Shanghai, China. The research team used NVivo 12.0 software. Giorgi’s phenomenological analysis method was used to analyse the interview data.

**Results:**

The interview results were divided into two categories in terms of patients’ experiences of FT and their coping strategies. Nine subthemes were constructed. The experience category included four subthemes: (1) taking on multifaceted economic pressure, (2) dual choice of treatment, (3) decline in material living standards, and (4) suffering from negative emotions such as anxiety and depression. The coping strategy category included five subthemes: (1) reducing expenses, (2) improving living habits, (3) proactive participation in medical decision-making, (4) making a job position choice, and (5) seeking social support.

**Conclusions:**

FT in young and middle-aged patients with stroke, which affected their physical and mental health, led them to implement strategies for dealing with FT. The Chinese government needs to broaden the reach of health insurance coverage and advance the fairness of healthcare policies. Healthcare professionals must pay active attention to FT in such patients in terms of strengthening their health education and considering their needs and preferences. Patients need to improve their sense of self-efficacy, actively reintegrate into society, and adhere to rehabilitation and treatment. Individuals at a high risk of stroke are recommended to purchase health insurance. Multifaceted efforts are needed to reduce the impact of FT in young and middle-aged patients with stroke.

**Supplementary Information:**

The online version contains supplementary material available at 10.1186/s12913-023-10457-z.

## Introduction

Stroke poses a global health challenge, ranking as the second leading cause of death and the third leading cause of death and disability combined in 2019 [1]. It is also a prominent cause of mortality in China. In 2020, there were 17.8 million reported stroke cases, with 3.4 million being new occurrences and 2.3 million resulting in stroke-related fatalities among adults aged 40 and older [2]. The increasing incidence of stroke among young adults can be attributed to several factors, such as the earlier onset of traditional vascular risk factors and an elevated prevalence of modifiable lifestyle risk factors like reduced physical activity or substance abuse [3]. Between 1990 and 2019, the prevalence rate increased by 22.0% and the incidence rate increased by 15.0% among individuals under the age of 70 [1]. Over the same time frame, there was a general increase in the hospitalization rate for stroke per 10,000 individuals among young and midlife adults (from 31.6 to 33.3) and a decrease among older adults [4].

Given that stroke frequently results in disability, this pattern presents a substantial challenge to economic stability, particularly in developing nations [3]. The expenses associated with stroke treatment and rehabilitation place a significant financial strain on patients, with estimates ranging from $1,809.51 to $325,108.84, depending on the severity of the condition [5]. Additionally, the one-year cost for initiating post-stroke rehabilitation stands at $70,601 for inpatients and $27,473 for outpatients [6]. The inflation-adjusted mean cost for inpatients has steadily increased, with an average annual growth rate of 2.44% among young people, 1.72% among middle-aged adults, and 1.45% among older adults, owing to the higher use of healthcare resources [4]. As a result, the economic burden of hospitalization and caregiving expenses for young and middle-aged stroke patients is rising when compared to their older counterparts [4]. Rutten’s study indicated that younger stroke patients had a 2–3 times higher risk of unemployment after eight years of follow-up [7]. Young and middle-aged patients are often the primary income earners in their families, and the reduction in financial income caused by unemployment adds to their financial and psychological burdens. Such costs, with the potential to impact health outcomes and induce financial and psychological distress in patients, have been referred to as financial toxicity (FT) [8].

Coined by Zafar in 2013, this term has subsequently been described as the ‘objective financial burden’ and ‘subjective financial distress’, both of which have adverse effects on patients’ health outcomes [9]. The objective economic burden primarily stems from the direct or indirect costs of treatment, while subjective economic distress includes material, psychological, and behavioural aspects. Material distress manifests as the accumulation of treatment expenses and income reduction, while psychological distress manifests as patients’ concerns over managing available assets, leading to anxiety and discomfort [10]. Apart from exacerbating symptoms, FT reduces the inclination to make payments and constrains adherence to treatment and follow-up schemes [11–13]. Studies have shown that FT leads to a threefold increase in the likelihood of emotional distress, encompassing conditions like depression and anxiety disorders, among patients [11]. Consequently, there is a pressing necessity to alleviate FT in patients.

FT has been considered in relation to patients with cancer as well in those with chronic diseases such as stroke and diabetes [14, 15]. Two nationally representative samples in the United States were obtained by analysing the National Health Interview Survey (2011–2017) and Medical Expenditure Panel Survey (2011–2016), with younger patients with stroke found to be more likely to report manifestations of FT, such as cost-related non-adherence to medication, and twice as likely to face difficulties in paying their medical bills than those without stroke [14]. A study from Xinjiang, China, using comprehensive scores for FT based on patient-reported outcome measures, found that high levels of FT were prevalent among patients with stroke [16]. Although published studies on FT in patients with stroke can enhance understanding of the current status of FT in such patients, the situation regarding the specific impact of FT on patients’ treatment, work, and life, as well as whether and what kind of coping strategies such patients adopt in the face of FT, remains unclear.

The FT experiences of older adult patients with cancer and their family members have been investigated using qualitative research methods [17]. That study offers valuable insights for the treatment of these patients and the implementation of intervention strategies to alleviate FT. Qualitative research can help researchers gain insight into the patient experience and appreciate the impact of FT on patients from the patient’s perspective [18]. Semi-structured interviews have also been employed to provide a flexible framework for delving into patients’ thoughts and experiences through follow-up questions [18]. Therefore, this qualitative study aimed to understand the FT experiences and coping measures of young and middle-aged patients with stroke, with the intention of aiding in the development of strategies for mitigating FT.

## Methods

### Study setting and participants

The participants were recruited from the Department of Neurology at a hospital in Shanghai, China. Purposive sampling was used to recruit the participants. Inclusion criteria were as follows: (1) patients diagnosed with stroke using computed tomography or magnetic resonance imaging; (2) patients aged 18 years and older and 59 years and younger [19]; and (3) patients with sufficient language skills to participate in an interview voluntarily. The exclusion criteria were as follows: (1) patients who were near to death, and (2) patients who asked to withdraw before interviewing was complete. Participants were enrolled continuously until data saturation was achieved or until no further new information emerged during the interviews [20].

### Data collection

Purposively sampled young and middle-aged stroke patients were subjected to semi-structured one-time interviews conducted from October 2022 to March 2023. According to the purpose of the study, the research team initially drew up the interview outline after discussion with reference to Su’s [17] and Schröder’s [21] studies, and selected three patients for pre-interviews. Pre-interviews were conducted to check whether the content of the interview outline could be understood by the patients and whether the purpose of the interview could be achieved to finalise the content of the interview outline. The results of the pre-interviews were excluded from the outcome analysis. The interview outlines were revised in the final version, appended as supplementary file (Table S1). The interview guide encompassed the following areas: (1) the financial status both pre and post-stroke; (2) the consequences of financial alterations on employment, daily life, and medical treatment; and (3) strategies to cope with financial change. The first researcher interviewed each participant. As a graduate student, she had acquired comprehensive skills in qualitative research methodologies. In her clinical practice, she interacts with patients in a friendly manner and builds a strong rapport that enables her to conduct interviews autonomously. Prior to the interviews, the participants gave their informed consent, and the entire process was audio-recorded. To ensure a private and noise-free setting, all interviews were conducted in a dedicated conference room, respecting patient confidentiality. When the patient’s physical functional status was good, the patient was interviewed alone. When the patient’s modified Rankin Scale (mRS) score was greater than or equal to 3, a caregiver accompanied the patient to facilitate care of the patient. Caregivers did not make any statements. All participants were interviewed once for 40 min to 1 h. While this research was carried out within one tertiary hospital in Shanghai, we also included young and middle-aged stroke patients from various provinces and cities who were hospitalised in this hospital, including those from Jiangsu, Anhui, and other regions.

### Data analysis

All transcripts were imported into NVivo 12.0, a software application used for qualitative data analysis. Two researchers with experience in qualitative research and who were familiar with FT actively participated in the data analysis. To safeguard patient privacy, the results were anonymised, and the interviewer’s name was replaced with a numerical identifier. The data analysis was conducted using Giorgi’s phenomenological analysis approach [22], adhering to the following methodological steps: (1) Listening and Reading: Thoroughly reviewing audio recordings and textual materials, attentively combining them to gain a comprehensive understanding of the data. (2) Data Segmentation: Segmenting the data into distinct sections, with a specific focus on the research subjects. This segmentation involved using professional sensitivity to identify “meaning units.” (3) Organization and Description of Raw Data: Ensuring the absence of repeated “meaning units,” providing clarification and elaboration on the relationships between these units and their relevance to the overall context. These meaning units were synthesized to create a coherent narrative. (4) Description of the Phenomenon’s Structure: Compiling all meaning units to articulate the fundamental essence of individual experiences. (5)Development of an Overarching Structural Description: Synthesizing specific structural descriptions derived from individual cases to formulate a comprehensive structural concept.

## Results

**Fig. 1:**
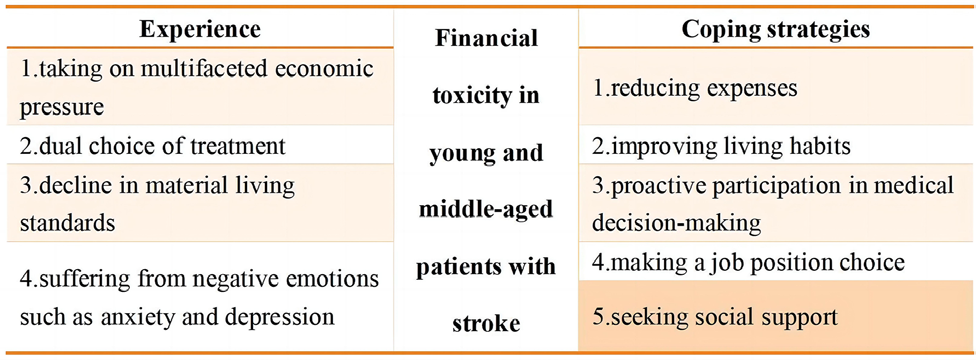
Themes and subthemes from the interviews

A total of 21 eligible patients with stroke, aged between 18 and 59, participated in the interviews and were assigned numerical labels (P1-P21) to protect their identities. Table 1 displays the participants’ demographic information. The analysis of the interviews revealed two main thematic categories, further divided into nine sub-thematic categories (Fig. 1).

**Table 1:** Sociodemographic characteristics of the participants (n = 21)

Code	Age	Gender	Types of stroke	Number of stroke episodes	mRS	Level of education	Marital status	Medical insurance	Permanent residence	Working condition	Number of children	Per capita monthly household income (Yuan)
P1	43	F*	IS*	First	1	College graduation	Married	Self-Paying	Jordan	Sickness unemployment	0	P*>10,000
P2	59	M*	IS	First	1	Senior high school	Married	UEBMI*	Shanghai	Retire	2	3000<P ≤ 5000
P3	59	F	HS*	First	0	Junior high school	Married	NRCMI*	Anhui	Unemployed	3	P ≤ 1000
P4	49	F	IS	First	1	Primary school	Married	NRCMI	Jiangxi	Sickness unemployment	5	3000<P ≤ 5000
P5	50	M	IS	First	2	Senior high school	Married	UEBMI	Shanghai	Leave for sickness	1	5000<P ≤ 10,000
P6	48	M	IS	First	2	Primary school	Married	URBMI*	Shanghai	Sickness unemployment	1	3000<P ≤ 5000
P7	59	F	IS	First	2	Senior high school	Married	UEBMI	Shanghai	Retire	1	5000<P ≤ 10,000
P8	39	M	HS	First	1	Junior college	Married	UEBMI	Shanghai	Leave for sickness	0	5000<P ≤ 10,000
P9	59	M	IS	Relapse	1	Senior high school	Married	URBMI	Henan	Self-support	1	P ≤ 1000
P10	43	M	IS	First	2	Junior high school	Married	NRCMI	Shanghai	Leave for sickness	2	3000<P ≤ 5000
P11	54	M	IS	Relapse	3	Junior high school	Single	URBMI	Shanghai	Unemployed	0	1000<P ≤ 3000
P12	55	M	IS	First	0	Junior high school	Married	UEBMI	Shanghai	Full-time	1	3000<P ≤ 5000
P13	48	M	IS	Relapse	1	Junior high school	Single	NRCMI	Jiangsu	Sickness unemployment	0	3000<P ≤ 5000
P14	46	M	IS	Relapse	2	Senior high school	Divorce	URBMI	Henan	Leave for sickness	0	5000<P ≤ 10,000
P15	49	F	IS	First	1	Junior high school	Married	URBMI	Shanghai	Unemployed	1	3000<P ≤ 5000
P16	51	M	IS	First	0	Senior high school	Married	NRCMI	Shanghai	Self-support	1	5000<P ≤ 10,000
P17	59	M	IS	Relapse	2	Junior high school	Married	NRCMI	Jiangsu	Unemployed	1	3000<P ≤ 5000
P18	57	F	HS	First	1	Primary school	Married	NRCMI	Jiangsu	Unemployed	2	1000<P ≤ 3000
P19	59	M	IS	Relapse	4	Junior high school	Married	UEBMI	Shanghai	Retire	1	5000<P ≤ 10,000
P20	56	M	HS	Relapse	3	Primary school	Married	UEBMI	Shanghai	Unemployed	1	1000<P ≤ 3000
P21	58	F	IS	First	2	Junior high school	Married	URBMI	Shanghai	Retire	2	3000<P ≤ 5000

*Note: *F* female, *M* male, *HS* haemorrhagic stroke, *IS* ischemic stroke, *UEBMI* urban employee basic medical insurance, *URBMI* urban resident basic medical insurance, *NRCMI* new rural cooperative medical insurance, *P* per capita monthly household income (Yuan)

### Experience of FT

#### Taking on multifaceted economic pressure

The young and middle-aged patients with stroke frequently experienced financial strain due to the expenses associated with stroke treatment, rehabilitation, and caregiving. Hospitalized patients, in particular, faced financial challenges brought on by the costs of tests, treatments, and medications.

This is the second time I’ve been hospitalised, and the cost of this hospitalisation is more than what I spent on my first hospitalisation for a stroke six months ago, which puts a lot of pressure on me. (P11)

Stroke continues to be a major cause of adult disability, with a growing need for stroke rehabilitation services. Nonetheless, as highlighted by certain patients, rehabilitation can be costly and is frequently not covered by health insurance.

I’m going straight to a rehab hospital for recovery after I’m discharged from the hospital, which will be a significant expense. (P14)

In addition, patients with severe functional impairment often required care from family members or staff of professional organisations. Family care reduces part of the family income, and the cost of care in specialised institutions puts financial pressure on such patients.

Now that I can’t walk, I have to rely on my children to take care of me in the hospital, which temporarily affects my children’s work, which in turn reduces the family’s source of income. (P19)

I’m going to a nursing home alone after this illness, I don’t want to involve my children in taking care of me, now I have some money left but I don’t know what to do when it runs out. (P21)

#### Dual choice of treatment

##### Actively seeking treatment

Some patients wanted to recover their physical condition faster by complying with professional treatment so that they could return to society faster.

I am actively working with my doctors on my treatment, and no matter how much it costs, my health is the most important thing in my opinion. (P7)

My hands are a bit numb at the moment. I would prefer to extend my hospital stay for a few more days to ensure a better recovery. (P8)

##### Intention to forgo follow-up treatment

Some patients affected by FT forwent specialised rehabilitation treatment and postponed follow-up reviews.

I don’t feel symptomatic at the moment, and I probably won’t be coming in for a review that early after I’m discharged. (P3)

I had to recover at home because I didn’t have big enough savings. (P20)

#### Decline in material living standards

##### Changing recreational activities and living a less satisfying life

The young and middle-aged patients with stroke were usually the mainstay of their families, with responsibility for supporting older adult family members as well their children. Some had to give up some of their former recreational activities because of the financial pressure caused by their illness.

I used to travel extensively, but now my health has deteriorated, and the uncertainty of recovery costs may limit my future outings. (P5)

##### Restricted basic living conditions

For the patients on average incomes, their basic living conditions were are also somewhat restricted.

Our house is far away from the hospital and for convenience before I took a taxi to the hospital which cost several hundred dollars. I should take the subway for a review afterward so I can save a little money. (P16)

Due to our limited financial situation…… I am even more hesitant to incur expenses following my illness. (P9)

#### Suffering from negative emotions such as anxiety and depression

Owing to the substantial financial burden associated with stroke, patients exhibited varying levels of psychological stress and apprehension regarding future treatment expenses during the interviews. Several patients described experiencing depressive symptoms, primarily manifesting as sleep disturbances and social withdrawal.

I always thought that stroke was a disease of the elderly. Now that I am young, the cost of medicine and review is a burden to me. It causes me to have frequent insomnia and no energy during the day. (P6)

Before that I liked to square dance with my friends. After I got sick, I didn’t feel like dancing when I thought I would have to spend a lot of money on treatment in the future, and gradually I didn’t have much contact with my former friends. (P15)

In addition, their illness generated concerns and anxiety about returning to work in those who were still working.

I fear that I might lose my ability to return to work if I continue on leave. The thought of it makes me anxious. How can I receive treatment without financial means? (P5)

### Coping strategies

#### Reducing expenses

Managing FT frequently involved cutting down on expenses. Some of the patients indicated that they made conscious efforts to reduce spending on rehabilitation and living expenses.

While I still experience some leg symptoms, I lack the means to attend a rehabilitation hospital, so I engage in self-guided exercises and rehabilitation at home. (P13)

There is a way to live with more money and a way to live with less money. Now for treatment, I have to spend less on my daily expenses. (P2)

#### Improving living habits

Unhealthy living habits are risk factors for stroke. Some of the patients said that this illness had made them realise their own inadequacies and that they needed to adjust their living habits to cope with the disease positively so as to minimise the likelihood of increased hospitalisation and medical expenses owing to disease recurrence.

I had the habit of smoking and drinking before, I didn’t realize that I would suffer from stroke at such a young age, I still need to pay attention in the future and try to adjust my life habits. (P8)

After I came home, I preferred to play mahjong and usually sat for hours without activity, so I still need to exercise more in the future to prevent the disease from progressing. (P7)

#### Proactive participation in medical decision-making

Owing to FT, some of the hospitalised patients indicated that they would consider the doctor’s recommendations and their own conditions and determine whether they really needed to undergo the relevant treatments and tests. In addition, they expressed some of their thoughts with the doctors.

The doctor suggested I need some kind of brain surgery and felt it would cost a lot of money. After thinking about it, I decided to go home and take my medication and not have the surgery. (P4)

I would check some medical information from the internet myself to find out if I need all the prescriptions that my doctor has prescribed. (P8)

I told the doctor I hoped he would prescribe more medication. The cost of the medication was included in the hospitalisation costs to be reimbursed together. (P16)

#### Making a job position choice

Owing to FT, some of the patients with milder symptoms were motivated to return to their former jobs and their workplaces, which were viewed as providing support.

Once my sick leave is over and I’ve recovered from my illness, I’ll be working as soon as I can, otherwise I’ll have no income after my savings are spent. My workplace also supports me to go back to work when I’m well enough. (P10)

Some interviewees mentioned that they may not be able to adapt to the intensity of their previous job because of the decline in physical functioning as a result of stroke. To maintain a regular source of income, they were prepared to voluntarily change their work roles according to their physical condition.

I worked on construction sites before, all heavy manual labour, and after this illness, I can’t do heavy manual labour anymore, so I’ll have to do some helper work from now on. (13)

When my body slowly recovers, I’ll go back to getting some hourly work to supplement my income. (P18)

#### Seeking social support

##### Seeking support from family members

When the patients did not have enough savings to cope with FT, they often suffered from negative emotions such as anxiety, and some interviewees indicated that they obtained support from family members.

I currently rely on my sister for various things. She not only takes care of me but also provides some financial assistance. (P11)

She (my spouse) has to go out and get a job to support the family and she is still very supportive and takes care of my emotions. (P16)

##### Seeking support from the community and the government

Some of the participants indicated that they would like to receive help from medical staff and would seek support from the community after discharge from the hospital.

We would seek help from the community, they have care policies for older people like us. (P20)

I saw a list of daily expenses at the nurse’s station, but I don’t know the exact reimbursement. I hope the staff can inform me of the reimbursement policy to facilitate my financial planning. (P8)

Some interviewees mentioned that the current health insurance reimbursement policy needed to be optimised to extend health insurance coverage and alleviate patients’ FT.

It seems that rehabilitation, review and medical expenses after discharge cannot be reimbursed, and it is hoped that in future health insurance can reimburse the expenses of follow-up related treatments after discharge. (P1)

## Discussion

This study delved into the experiences of FT in young and middle-aged patients with stroke and the coping strategies they applied. Stroke often led to functional impairments that increased the cost of treatment, rehabilitation, and care. In addition, the inability to continue working during treatment generated FT in young and middle-aged patients with stroke. Relevant studies have confirmed that 81.18% of patients with stroke have varying degrees of FT, with the level of FT being higher in young and middle-aged patients with stroke [16]. One study indicated that the expenses related to stroke treatment exhibited no substantial reduction either at the time of discharge or at the three- or six-month marks, with the highest costs observed during the initial six months [23]. In this study, the patients stated that they bore the financial pressure of treatment and subsequent rehabilitation and care, an issue that requires urgent attention from medical professionals.

FT had a dual effect on patient care. On the one hand, FT prompted an active response to the disease in terms of wanting to recover as quickly as possible; on the other hand, there was an intention to delay seeking treatment owing to FT. Some patients expressed that they would comply with treatment to improve their symptoms. Positive coping with a disease can improve patients’ health behaviours, thereby facilitating recovery while reducing negative emotions [24]. However, some patients expressed contrasting intentions, such as giving up or postponing reviews because of the high cost. Studies have reported that the economic status and educational level of patients with stroke affects their medication literacy and adherence to rehabilitation [25, 26], such that the lower the annual household income and level of education, the more likely patients were to have treatment non-adherence. Non-compliance with treatment regimens can have adverse effects on patients’ overall treatment outcomes and exacerbate FT. Healthcare providers should enhance patient education and underscore the significance of adhering to medication and rehabilitation plans.

FT encompasses not only an evident economic burden but also subjective financial distress. In light of this research, it becomes evident that the substantial economic load associated with stroke treatment and rehabilitation leads to symptoms such as anxiety, depression, and apprehension about the future among patients. Other research has associated the financial status of post-stroke patients with the extent of depression and anxiety, with the greatest effect on alleviating depression severity [27]. These findings also resonate with studies involving cancer patients in China, where post-stroke anxiety and depression hinder the progress of stroke recovery and rehabilitation [28]. Moreover, middle-aged and young patients with stroke typically serve as the primary financial contributors to their families before falling ill. However, due to leaves of absence or unemployment stemming from their condition, and for those with severe symptoms necessitating care, patients may grapple with the temporary disruption of their roles. This disruption further intensifies negative emotions, detrimentally affecting their quality of life and prognosis. To address this, healthcare providers should implement effective psychological interventions to help patients with stroke manage the psychological burden posed by the disease. Patients with more severe symptoms can experience a reduction in FT by actively participating in psychological counselling and adhering to their treatment regimen.

Stroke often imposes a significant financial burden on patients and their families. During the interviews, a majority of the patients reported employing various strategies to address FT, either already implemented or planned for the future. This involved taking steps to reduce rehabilitation and living costs, as evidenced by intentional measures to reduce costs, such as foregoing rehabilitation in specialised institutions and reducing the number of visits to the doctor. Studies on FT in patients with cancer have found that patients appeared to curtail treatment and living costs owing to FT [21], which is similar to the results of this study. Some patients mentioned that they could improve their lifestyle habits to cope with FT. Harshfield’s findings supported the hypothesis that lower levels of education, increased smoking, and obesity increase the risk of stroke [29]. Good dietary and lifestyle habits promote recovery and alleviate treatment costs in the long run. Therefore, it is recommended that healthcare professionals follow up with patients regularly, especially within six months after discharge, to remind them of good treatment adherence and lifestyle habits.

In addition, some patients expressed a willingness to actively participate in medical decision-making, which would involve determining the appropriateness of treatment options based on their own values and needs. High-quality and effective provision of health information plays a crucial role in enabling patients to actively engage in decision-making and healthcare, empowering them to effectively manage their chronic conditions. Simultaneously, healthcare professionals should proactively listen to patients’ needs. Research has indicated that patients desire discussions with healthcare providers about healthcare costs, with 74% believing that such discussions should be initiated by doctors [30]. Physicians need to consider the impact of medical decision options on the likelihood of FT as experienced by patients [31]. Therefore, healthcare professionals should establish effective communication bridges and attend to patients’ concerns and preferences to ensure that treatment plans align with their actual needs and expectations.

Moreover, some patients with very mild symptoms reported that they could cope with FT by returning to work. Carlson suggested that encouraging the social reintegration of cancer patients can serve as an effective coping mechanism for dealing with FT [32]. Similarly, for adults recovering from a stroke, a common objective is the resumption of work, a goal that can substantially enhance their overall quality of life [33]. After resuming work, certain stroke patients encounter fatigue [34]. In this study, certain respondents indicated that they would give due consideration to their symptoms and make positive adjustments in their jobs. This showed that these patients were prepared to actively seek to reintegrate into society. Hence, it is essential for healthcare professionals to initially assess a patient’s potential for societal reintegration using the Return-to-Work Self-efficacy Scale [35]. Patients should then be provided with relevant employment information to enhance their ability to participate in society and obtain suitable employment.

These types of patients tend to live for long periods of time with their families; therefore, patients who bear the burden of FT usually seek support from their families. In this study, certain patients motivated their spouses to seek employment in order to augment their family’s income. This finding was inconsistent with the results reported by Jeon et al. [36]. Nonetheless, this research concentrated on young and middle-aged stroke patients, among whom there were individuals experiencing their first stroke, presenting mild symptoms, and not requiring caregiving; therefore, it was more likely that their spouses might work to increase their income. Liu et al. reported that good family support can reduce the sense of shame in patients with stroke, facilitating positive perceptions and their ability to cope with the disease [37]. Some participants stated that their family members provided them with strong support and care. Future research should focus on the strategies and challenges encountered by patients with stroke and their families as they navigate the complexities of FT. Such research is likely to enhance comprehension, prevention, and mitigation of FT experienced by patients and their families throughout the course of treatment.

During the interviews, the patients mentioned that knowing their medical costs through cost lists available at nursing stations helped them with financial planning. However, some were unsure about the health insurance and reimbursement rates. The expenses incurred by stroke patients in the six months following discharge have been reported not to exhibit a significant decrease compared to the costs during hospitalisation [23]. However, only hospitalisation only hospitalization costs are eligible for reimbursement, which further worsens the FT experienced by stroke patients. Several participants expressed the desire for government intervention to expand health insurance coverage. Yang highlighted substantial disparities in healthcare utilization and medical expenditures among stroke patients both within and across regions in China [38]. Given this situation, the government in China needs to better integrate the national health insurance system, improve its overall structure, broaden health insurance coverage, and ensure equitable welfare and financial protection for patients with stroke across diverse regions.

Most adults in China are covered by health insurance, and the rate of uninsured individuals is as low as 3.15% [39]. It’s noteworthy that one self-paying patient, previously residing abroad for an extended period, contributed to our study’s responses. Existing research indicates that self-funded patients, individuals with more severe disease symptoms, and those with lower socioeconomic status experience increased levels of FT [40–42]. Furthermore, a relevant study revealed that inpatients covered by employee health insurance received reimbursements but still incurred significant out-of-pocket expenses, which constituted 43.54% of their total healthcare costs, thereby exerting financial strain [43]. The adoption of health insurance has demonstrated a remarkable capacity to enhance residents’ overall health status, with rural populations benefiting more than their urban counterparts [44]. Therefore, individuals at heightened risk of cardiovascular disease are strongly encouraged to explore health insurance options.

## Strengths and limitations of this study

To the best of our knowledge, this study represents the initial investigation into the experiences and coping mechanisms related to FT in young and middle-aged stroke patients. In this study, we enrolled 21 young and middle-aged stroke patients with diverse disease types, durations, severity, health insurance coverage, and family income levels. It is essential to underscore that our interview approach was dedicated to gaining profound insights into the participants’ perspectives. Consequently, individuals who were unable to comprehend the interview or express coherent responses, such as those concurrently experiencing severe dementia or aphasia due to stroke, were excluded from the study. Therefore, the findings of this research are particularly relevant to young and middle-aged stroke patients who exhibit good comprehension and communication abilities.

## Conclusion

This study delved into the encounters with FT and the corresponding coping approaches in young and middle-aged stroke patients. These patients reported experiencing various economic pressures, and that FT had affected their treatment, quality of life, and emotions. Most patients had adopted specific strategies to cope with FT, including reducing expenses, improving lifestyle habits, actively participating in medical decision-making, choosing more suitable jobs or modifying their current job role, and seeking social support. According to the findings of this study, there is a need for the Chinese government to broaden the coverage of health insurance and advance fairness in health insurance policies. Medical workers should pay more attention to FT, strengthen patient health education, actively communicate with patients, and fully consider patient preferences and needs. Patients should improve their self-efficacy, increase their adherence to rehabilitation and treatment, actively reintegrate into society, and seek social support when necessary. Individuals at an increased risk of stroke are encouraged to invest in health insurance. Subsequent research endeavors should conduct additional interviews with patients’ spouses or primary caregivers, and delve into additional approaches to alleviate stroke-induced FT within the family context.

### Electronic supplementary material

Below is the link to the electronic supplementary material.

**Supplementary Material 1:** Interview Guide

## Data Availability

The datasets utilized and/or examined in this study can be obtained from the corresponding author upon making a reasonable request.

## List of abbreviations

FT: financial toxicity
mRS: modified Rankin Scale
