# Supplementary material for: Experience of financial toxicity and coping strategies in young and middle-aged patients with stroke: a qualitative study
Source: BMC Health Serv Res. 2024 Jan 17;24:94. doi: 10.1186/s12913-023-10457-z (PMC10795406; doi:10.1186/s12913-023-10457-z)
Supplement: Supplementary file 1 — Supplementary Material 1: Interview Guide [file 12913_2023_10457_MOESM1_ESM.docx]

**Table S1: Interview Guide**

| No. | Question |
| --- | --- |
| 1 | Have you felt financial pressure since you were diagnosed with a stroke? |
| 2 | Can you talk about the family finances before and after the stroke diagnosis? |
| 3 | How has your financial situation changed since your stroke diagnosis? |
| 4 | How has the financial change brought about by stroke affected your work, life, and treatment? |
| 5 | How have you coped with these financial changes? |
| 6 | What support are you looking for to help you cope with this change in your financial  changes? |
